# The Treatment of Patients after Abdominal Section

**Published:** 1909-08-21

**Authors:** William Turner


### Hospital Clinics.

### THE TREATMENT OF PATIENTS AFTER ABDOMINAL SECTION.

By WILLIAM TURNER, M.S., F.R.C.S.

(A Lecture delivered at the Seamen's Hospital, Greenwich.)

Gentlemen,—Before I speak of the after-treatment of a patient who has had a laparotomy performed, I must call your attention to the fact that the importance of the care and preparation of the patient, the kind and method of the anæsthetic, and the care of the patient during the operation cannot be over-estimated. Take a patient who has had a clean operation performed, the abdominal wall sewn up, and the bowel not injured—I emphasise that because it is most important—such as for the removal of a fibroid, as a typical example of the kind of case I am referring to. The patient is put to bed, preferably on the back; the head is turned to one side, a pillow is put under the opposite shoulder and another under the knees. A cradle may or may not be used, according to the sensibility of the patient and the type of the operation. A nurse keeps the head over if vomiting occurs, and her other hand should be placed over the bandages to support the wound when straining and vomiting occur; it is sometimes very useful to mark the outside of the bandage with a blue pencil to indicate the position of the wound beneath.

Unless there is some definite indication there is no need to do anything more until the patient comes out of the anæsthetic, when the two symptoms, pain and restlessness, are the ones to be treated. In nearly every case morphia is the best agent here to employ. Doses of not more than $\frac{1}{4}$ grain of morphia can be given to a patient, either just before the anæsthetic is administered, before the patient is moved to bed, or just when coming round from the anæsthetic. The severity of the manipulations and the general condition of the patient determine the best time. It is frequently necessary to delay the administration of morphia until the patient has spoken after the anæsthetic on account of the anxiety of the friends. A disadvantage in giving it early is that it often delays and thereby aggravates the anæsthetic vomiting; the saliva, ethereal vapour, and mucus remain in the stomach, and a considerable amount of gastritis is the result. Perhaps the best routine method is to allow the patient to get the early vomiting over, and then, if uncomfortable, to give morphia. The dose must be quite small, for there is no doubt that morphia increases distension and flatulence; do not repeat it unless absolutely necessary. If the morphia is combined with atropine or strychnine, it considerably obviates this distension; but the disadvantage of atropine is that it increases the patient's thirst. Then the bladder must be attended to, and the patient should pass water within about eight hours of the operation. Some patients can be left longer, even to twenty-four hours; but I think it is unwise, and that the catheter should be used if it is not passed in fifteen hours. In women, especially pelvic cases, this is very important; it is better to run the risk of passing a catheter than to have an atonic bladder wall, and that may even cause death from suppression. The restlessness and sleeplessness on the first night after operation are often due to this cause, and relief and sleep follow immediately on the passage of a catheter. When the urine is scanty and of very high specific gravity, saline solution with 20 or 30 grains of acetate of potash may be given per rectum. Uncomfortable bandages, or a full bladder, or an awkward position in bed, may account for loss of sleep on the first night. Or the patient may be a drug-taker, either alcohol, morphia, or some hypnotic, and this makes the treatment very difficult indeed.

Is change of position harmful in these cases of discomfort? I do not think so if it is done to the patient and not by him. To be rolled a little on one side often gives relief, or a pillow under the shoulders, or a small one under the middle of the back may help. If these means do not produce sleep, you are bound to try drugs. Thirty grains of bromide given per rectum, dissolved in 3 oz. of water very often acts well. Other drugs which may be given in exactly the same way are trional, chloralamide, chloral, or a mixture of chloral and bromide. If these fail, a morphia suppository, which does not cause anything like the effect on the general condition, may be very useful indeed. Where the patient is very ill from peritonitis, strychnine has an undoubted effect in causing sleep, though it is really given to improve the pulse.

We next come to the question of feeding. As a regular rule one says no food for twelve hours. A little hot water to sip and wash the mouth out with. If great thirst is complained of, saline solution per rectum is the best antidote. After twelve hours the water may be increased in quantity to 2 ozs. at a time; then albumin water is given alternately with the water, always providing that vomiting is not going on. If the patient is still vomiting, nothing in the way of food for two hours. On the second day, if the patient is not vomiting, tea, coffee, meat juice, beef tea, and jelly may be allowed. But, unless the patient is a large milk taker, it is much wiser to withhold milk. This is absolutely contrary to all the teaching in hospitals. It is better to withhold milk until the bowels have acted, as the increase in distension is often most marked when patients have milk. If milk is given, it may be either peptonised (but it is very nasty) or diluted with barley water; then the dilution is gradually diminished until milk is given by itself. On the third day the patient may have toast, which I think is very good, and tea and an egg, a little pounded fish and chicken, as well as the things already mentioned. After that there may be a steady increase to light convalescent diet, usually with a little fruit; and after ten days the patient may be having practically ordinary diet. Pastry and green vegetables are not usually well taken till then. You will notice I have not mentioned anything about rectal feeding; but in an uncomplicated case it is absolutely absurd to feed per rectum.

It is wise to get the bowels to act on the third or fourth day. I usually choose the third night for giving an aperient where is is safe to administer one. I never administer an aperient where the bowel has been opened at the operation unless peritonitis is present or imminent. Enemata are the rule in these cases; either simple or with turpentine, or in more obstinate cases one consisting of olive oil, glycerine, and turpentine.

Rest is the most important thing in the treatment of these cases; and by giving an aperient the local rest of the part of the intestine which has been wounded is destroyed, and may prevent the adhesions which you want to form. In the ordinary way castor oil is the best aperient to give in the early morning. Where peritonitis is present or imminent, as after a severe operation for perforation of the appendix where the peritonitis is general, and on the third day there is considerable distension and hardness of the abdomen, nothing answers better than small doses of calomel, ½ grain every hour until ten doses have been given or the bowels have been moved, combining with this hypodermic injections of strychnine, 1-30 grain, or salicylate of eserine 1-100 grain, which has proved of service in some cases.

It is at about this time, the second or third day, that the bandages become tight from distension, the breathing may be hampered, and the patient may become very restless. Loosening the bandage is the treatment, and when the bowels act, it should be tightened up again.

The regular action of the bowels after the first time depends, again, upon the patient. Usually one finds that either small doses of cascara in women—men do not take it at all well, in my experience—or senna pods, liquorice powder, salines, or Apenta water will bring about the desired result. Some patients prefer not to take drugs, and then glycerine suppositories or ordinary enemata every morning are useful.

In the majority of cases it is wise to look at the wound on the fifth day, and remove and change the dressings, which again is contrary to some of the dicta. I think it is more important to make the patient comfortable at this stage than to mind about the risk of undoing the wound, which is nil in skilled hands. The moist hot skin of the back under the bandages and the skin between the legs can be thoroughly washed with spirit, and powdered (of course keeping the wound covered). Stitches which are too tight, or around which there is inflammation, can be taken out. A similar dressing to the original one is put on and the bandages reapplied. On the tenth day the superficial stitches can be removed, a layer of gauze spread well over the anterior wall of the abdomen, and fixed with collodion right down, so as to make a firm support to prevent stretching of the scar. When dry, the bandages are re-applied as before; and care must again be taken that while this is being done there shall be no straining, especially where the abdominal wall is sutured with one layer of stitches. All wounds are by no means firmly healed on the tenth day. If the stitches have been taken out and the patient suddenly strains, the wound may open with prolapse of the intestine. This is especially so in urgent abdominal operations, where the patient is ill either with acute intestinal obstruction, or intussusception, or something of the sort.

On the 18th day the patient may sit up in bed, may get up on the 21st, and may usually be walking about on the 24th. I do not believe in shortening these periods, as embolism, thrombosis, or stretching of the scar are likely to occur. Also I do not think many patients are mentally fit to go about until the end of the fourth week after a laparotomy.

In the late treatment there are two things in particular to be looked after: (1) the liability to hernia; (2) the liability to adhesions. The precautions against hernia are the accurate stitching of the wound (the material being quite aseptic), and the knots being not too tightly tied; preventing extra strain while vomiting, coughing, etc., by keeping the walls supported by a bandage and belt; and early exercises to get the muscles strong again, and the avoidance of sudden straining—in particular such straining as lifting up a heavy window, or picking up a large can full of water so as to pour it into a basin, or strapping up a heavy box. Adhesions occur when least expected, and it is almost impossible to foresee, in any given case, whether they are going to be present or not. As a rule we find that they are due to leaving a portion of the surface of the peritoneum uncovered, as often happens in the removal of an ovarian tumour or the removal of omentum. Much handling of the intestine may bruise the peritoneum over it; and antiseptics may irritate it and cause adhesions. Where, from any cause, there is a likelihood of adhesions, the injection of saline solution into the peritoneal cavity at the end of the operation is recommended, though not by all surgeons. Ensuring peristalsis is the best way of dealing with and preventing them during the first few months after the operation; and so constipation must be very carefully avoided.

I now come to some of the complications requiring treatment on their own account. Shock, if very severe, is best treated by the infusion of saline solution into a vein, with a drachm to the pint of solution of adrenalin chloride 1 in 1000. Two or three pints of this are injected into the vein, and repeated if necessary, more particularly if the shock is complicated with hæmorrhage. Also combine with this a small dose of morphia. This relieves restlessness, and has some effect on the shock itself. In slighter cases a similar injection of a pint at a time may be given every four hours per rectum. I do not give any stimulants unless the condition is very urgent. If shock is so urgent as to require stimulants before adrenalin solution can be absorbed, then ammonia to smell, brandy, ether, and strychnine hypodermically are probably the best things that can be given.

Now with regard to vomiting. The first vomiting, of course, is good for the patient. But when it goes on during the first twenty-four hours it may distress and weaken him very much. The cause is nearly always the anæsthetic. A large quantity of hot water, with a teaspoonful of bicarbonate of soda to the pint, will cause first vomiting and then relief. If still persisting, lavage by means of a stomach tube with saline solution, and leaving in a drachm of subnitrate of bismuth in suspension, may stop it. Other things that do good are a mustard leaf over the epigastrium, and heat. The Americans especially recommend ice on the abdomen, though we do not do it much over here. There is a list of drugs which begins at one end of the Pharmacopœia and ends at the other; practically every drug has been tried. Americans use inhalations of vinegar, and they speak highly of it. They also give salicylate of bismuth gr. x., calomel gr. $\frac{1}{2}$, and Dover's powder gr. $\frac{1}{4}$, by the mouth. I doubt whether ipecacuanha and calomel, with the patient vomiting from the anæsthetic, is likely to be of use unless you do also what we do, give small doses of morphia to quieten the patient for a time, and afterwards a little solid food and black coffee, to which may be added bromide of sodium gr. x. If vomiting persists to the second or third day you must feed the patient per rectum. I usually give injections of saline and nutrient enemata alternately every six hours, the latter of two ounces of peptonised milk, two ounces of beef tea, two eggs, and twenty grains bicarbonate of soda; which acts very well. After four of these nutrient enemata the bowel must be washed out; then start again. Brandy, of course, can be given either with the saline or with the nutrient enemata. If the vomiting increases on the third day, or recommences then, it must be looked upon as a symptom of severe import, usually due to intestinal obstruction, peritonitis, or, occasionally, uræmia.

Hiccough is occasionally very distressing in the early stages after laparotomy. I have seen many things tried for it, but there are three things which seem sometimes to help. One is to lay a spoon on the back of the tongue, as far back as possible, and keep it firmly pressed there. I think that is reflex. Spiritus etheris co. and dilute acetic acid have been recommended: a teaspoonful of each in an ounce of water. I do not think anyone knows the cause of hiccough.

Abdominal distension often requires treatment, and the passing of a rectal tube, turpentine or asafœtida enemas—if it were not for the smell I think the latter is preferable to the turpentine—and a firm bandage with light rubbing over the abdomen by the nurse, the regular administration of strychnine, or salicylate of eserine $\frac{1}{100}$ grain hypodermically three-hourly, sums up the treatment of a severe case. If there is no vomiting, but only distension, and there is no other contra-indication, an aperient can be given. Of the aperients, castor oil, calomel, or mag. sulph. may have the desired effect. If the distension is a symptom of peritonitis, then probably your only hope is to treat the peritonitis by operation first. And in a case like that, where there is extreme distension and peritonitis, the operative treatment entails opening and emptying the bowel, as well as treating the peritoneum.

When I spoke of sleeplessness before, it was particularly with regard to the first night. Some patients suffer from insomnia the whole of the time after an operation; and, of course, the only thing to do is to ascertain the cause and treat it. If it is due to drugs taken before, give doses of the drugs. If it does not appear to be due to anything definite, there are two things which may help. One is warm sponging by the nurse, and the other is facial massage. Otherwise give bromide per rectum, trional or chloralamid. In plain insomnia do not give morphia.

In the treatment of cases where drainage has to be allowed for general peritonitis, the sitting posture has proved to be of great advantage. Then the dressings have to be so arranged as to be easily removed, and they must be changed as often as necessary, usually daily, or even oftener. Plugs usually do not require removal until forty-eight hours after the operation, and then only part is taken out at a time if difficult to remove. Remember to be careful not to leave a piece in for good.

Drainage tubes can, as a rule, be replaced by plugs directly the discharge of pus ceases, usually, in the loins, on the third day. But the tube draining the pelvis must not be removed until nothing at all is got out of it on a piece of ribbon gauze passed down to the bottom. In a bad case such a tube may be in for a fortnight. These places in the abdominal wall where tubes have been are very liable to stretch and cause herniæ, and, unfortunately, may call for a secondary operation. But when the whole wound is left open and all goes quite well, secondary suture can be performed from the 10th to the 21st day. This may produce an excellent scar, with no tendency to hernia at all. In cases where the gall-bladder is drained, the tube can be either tied in with a purse-string suture, or, better, an opening left in the gall-bladder only large enough to take the tube; the tube can then be brought through the dressings and layers of many-tailed bandages and allowed to drain into a vessel by the side of the patient on the bed. Where a Paul's tube has been introduced into the bowel, a thin rubber tube, passing down to a vessel on the floor from the glass tube, is the easiest method, the glass tube itself passing through the dressings and bandages. Remember a Paul's tube cuts out of the bowel in from 24 to 48 hours, and then requires replacing. If the wound is high up in the small intestine the patient starves to death unless the obstruction is removed and the contents passed on again.

In operations on the stomach the after-treatment which I have given you must be modified somewhat, as nothing but a little water should be given for three days, and in some cases the interval should be longer. This, of course, entails rectal feeding for a much longer period than the first twenty-four hours.

Time does not permit me to deal further now with the various points connected with the nursing of these cases, or of the symptoms requiring treatment which mark the onset of complications. But I have tried to put before you the general lines to guide in the treatment of the bulk of the cases. Still, as is the case with all rules, they require to be applied with discretion, and occasionally with very considerable modification.

